# Implementation of a Test, Treat, and Prevent HIV program among men who have sex with men and transgender women in Thailand, 2015-2016

**DOI:** 10.1371/journal.pone.0201171

**Published:** 2018-07-25

**Authors:** Sumet Ongwandee, Cheewanan Lertpiriyasuwat, Thana Khawcharoenporn, Ploenchan Chetchotisak, Ekkachai Thiansukhon, Niramon Leerattanapetch, Banlang Leungwaranan, Chomnad Manopaiboon, Thanongsri Phoorisri, Prin Visavakum, Bongkoch Jetsawang, Monsicha Poolsawat, Somboon Nookhai, Monthinee Vasanti-Uppapokakorn, Samart Karuchit, Chonticha Kittinunvorakoon, Philip Mock, Dimitri Prybylski, Ake-Chittra Sukkul, Thierry Roels, Michael Martin

**Affiliations:** 1 Thailand Ministry of Public Health, Nonthaburi, Thailand; 2 Thammasat University Hospital, Patumthani, Thailand; 3 Srinagarind University Hospital, Khon Kaen, Thailand; 4 Udon Thani Hospital, Udon Thani, Thailand; 5 Khon Kaen Hospital, Khon Kaen, Thailand; 6 Lerdsin Hospital, Bangkok, Thailand; 7 U.S. Centers for Disease Control and Prevention, Division of HIV/AIDS and TB-Thailand, Nonthaburi, Thailand; University of Washington, UNITED STATES

## Abstract

**Introduction:**

Antiretroviral therapy reduces the risk of serious illness among people living with HIV and can prevent HIV transmission. We implemented a Test, Treat, and Prevent HIV Program among men who have sex with men (MSM) and transgender women at five hospitals in four provinces of Thailand to increase HIV testing, help those who test positive start antiretroviral therapy, and increase access to pre-exposure prophylaxis (PrEP).

**Methods:**

We implemented rapid HIV testing and trained staff on immediate antiretroviral initiation at the five hospitals and offered PrEP at two hospitals. We recruited MSM and transgender women who walked-in to clinics and used a peer-driven intervention to expand recruitment. We used logistic regression to determine factors associated with prevalent HIV infection and the decision to start antiretroviral therapy and PrEP.

**Results:**

During 2015 and 2016, 1880 people enrolled. Participants recruited by peers were younger (p<0.0001), less likely to be HIV-infected (p<0.0001), and those infected had higher CD4 counts (p = 0.04) than participants who walked-in to the clinics. Overall, 16% were HIV-positive: 18% of MSM and 9% of transgender women; 86% started antiretroviral therapy and 46% of eligible participants started PrEP. A higher proportion of participants at hospitals with one-stop HIV services started antiretroviral therapy than other hospitals. Participants who started PrEP were more likely to report sex with an HIV-infected partner (p = 0.002), receptive anal intercourse (p = 0.02), and receiving PrEP information from a hospital (p<0.0001).

**Conclusions:**

We implemented a Test, Treat, and Prevent HIV Program offering rapid HIV testing and immediate access to antiretroviral therapy and PrEP. Peer-driven recruitment reached people at high risk of HIV and people early in HIV illness, providing an opportunity to promote HIV prevention services including PrEP and early antiretroviral therapy. Sites with one-stop HIV services had a higher uptake of antiretroviral therapy and PrEP.

## Introduction

In Thailand, epidemic modeling suggests that 43% of new HIV infections in 2015 occurred among men who have sex with men (MSM)[1]; however, HIV testing among MSM remains low outside urban areas and has only increased in urban areas in recent years [2,3]. Thus, many MSM living with HIV infection do not know they are infected, delaying use of life-saving antiretroviral treatment (ART).

ART reduces the risk of serious illness among those infected with HIV regardless of CD4 count [4, 5] and ART can reduce the risk that people living with HIV (PLHIV) will transmit HIV to their sexual partners [6]. Randomized controlled trials have also shown that daily use of tenofovir or tenofovir-emtricitabine, HIV pre-exposure prophylaxis (PrEP), can reduce the risk of HIV infection among people who inject drugs (PWID)[7], heterosexual adults[8,9], and MSM [10].

Increasing HIV testing coverage, helping those who test positive initiate and stay on treatment, and providing HIV prevention services to those who test negative are important components of Thailand’s National Operational Plan for Ending AIDS 2015–2019 [11]. In 2014, Thailand issued national guidelines recommending ART for HIV-infected adults regardless of CD4 count and PrEP for people at high risk of HIV infection [12]. Thailand has a government supported universal health care program that includes ART for PLHIV but does not currently pay for PrEP [13].

Data on the knowledge and attitudes of MSM and transgender women about ART initiation and PrEP are lacking in many parts of Thailand. These data can help providers and public health officials tailor messages to improve uptake of ART and PrEP. In an effort to increase HIV testing coverage among MSM and transgender women, and to identify potential barriers to the immediate initiation of ART and the use of PrEP, we launched a Test, Treat, and Prevent HIV Program at five hospitals in four provinces of Thailand. Here, we describe participant recruitment and enrollment; knowledge and attitudes about HIV, ART, and PrEP; and factors associated with prevalent HIV infection, and ART and PrEP uptake.

## Methods

### Participant recruitment

We developed educational flip charts and brochures to inform potential participants about HIV, ART, and PrEP. We posted information about the study on a Thailand Ministry of Public Health managed web-site (http://www.buddystation.org/) and worked with local non-governmental organizations to inform MSM and transgender women about the study. Study staff were available at the sites to discuss the benefits of early ART and PrEP, enrollment criteria, and study procedures with individuals who walked-in seeking information about the program.

In an effort to increase HIV testing among people who did not regularly come to the clinics for services, we also used a peer-driven intervention adapted from respondent-driven sampling methodology to recruit participants [14–16]. Staff selected four enrolled participants (i.e., peer-recruiters) at each site to begin recruitment chains. Staff selected peer-recruiters based on their knowledge of HIV transmission and prevention and willingness to help others access HIV testing. Staff provided training about stigma and discrimination, study procedures, and the benefits and risks of HIV testing to peers. Standardized educational materials were provided to the peer-recruiters to use to recruit peers. Each recruiter was given three coupons to give to peers deemed likely to enroll in the study and received 100 baht ($3.00 U.S. dollars) compensation for each person who enrolled.

### Study sites

The Test, Treat, and Prevent HIV program was implemented in five hospital-based outpatient clinics. Khon Kaen Hospital, an 867-bed government hospital in Northeastern Thailand, Lerdsin Hospital, a 500-bed government hospital in central Bangkok, and Udon Thani Hospital, a 924-bed government hospital in Northeastern Thailand provide HIV counseling, testing, and diagnosis and treatment services in one clinic area. Srinagarind Hospital, a 1466-bed university hospital in Khon Kaen Province, and Thammasat Hospital, a 541-bed university hospital in Prathum Thani Province 20 miles north of Bangkok, provide HIV counseling and testing in one clinic site and laboratory and treatment services in different clinics at the hospitals. All hospitals implemented the Test and Treat Program and Lerdsin and Thammasat Hospitals also implemented PrEP.

### Enrollment and study procedures

We offered enrollment to Thai MSM and transgender women aged 18 years old and older, not known to be HIV-infected, who reported having anal intercourse with a male or transgender woman partner without using a condom in the 6 months before enrollment. Trained study staff explained the study purpose, procedures, discussed advantages and disadvantages of HIV testing and ART use, and confidentiality to potential participants in private areas of the clinics. Those who met the eligibility criteria and signed the consent form could enroll. Participants completed a standardized, tablet-based, questionnaire, and had HIV testing using a 3-test algorithm consistent with Thailand’s national HIV testing guidelines [17] at baseline; the questionnaire was repeated every 6 months for 18 months. We worked with hospital laboratories to ensure test results could be provided within two hours of testing. Participants with negative HIV test results were asked to return each 6 months for repeat HIV testing until they completed 18 months of follow-up. Participants with positive HIV test results were offered ART and followed every 6 months for 18 months.

At Lerdsin and Thammasat Hospitals, staff asked participants with negative HIV test results if they would be interested in learning about PrEP and willing to complete a questionnaire. We offered PrEP to participants who signed consent, had no signs or symptoms of acute HIV infection, and a creatinine clearance ≥60 ml/min [18]. Participants were offered PrEP for 12 months, given an information sheet listing dosing instructions and side effects, and provided adherence counseling and Hepatitis B virus surface antigen (HBsAg) and antibody (anti-HBV) testing. Participants who were HBsAg and anti-HBs negative, were advised to seek HBV vaccination and provided with information about HBV vaccination sites and referrals. Participants who were HBsAg positive and anti-HBs negative, were counseled on PrEP medicine HBV suppression and potential hepatic flares.

We asked participants who chose to take PrEP to return one month after starting PrEP for an HIV test. If no dose-limiting side effects or adherence problems were detected, we asked participants to return in two months and every 3 months thereafter for HIV testing, adherence and risk reduction counselling, and an assessment for adverse events. Creatinine clearance was checked at 6 and 12 months, and if the creatinine clearance was <60 ml/min, PrEP medication was stopped. Staff directed participants who wanted to continue PrEP after 12 months to clinics where they could buy PrEP medication.

### Analysis

We limited analyses to data and specimens collected at enrollment. We used logistic regression stratified by site to determine factors associated with prevalent HIV infection, the decision to start ART, or the decision to start PrEP. For analysis, we defined participants who identified as bisexual, gay, or heterosexual and reported sex with a man or transgender women in the 6 months before enrollment as MSM and participants who self-identified as transgender women as transgender women. Variables with a p-value <0.15 in bivariable analysis were evaluated in a multivariable model. We used SAS version 9.3 (SAS Institute, North Carolina, USA) for statistical analyses.

### Ethical review

This activity was approved by the Thailand Ministry of Public Health Ethical Review Committee and as a non-research program evaluation by the U.S. Centers for Disease Control and Prevention and the procedures followed were in compliance with the Helsinki Declaration.

## Results

### Participants

From April 2015 through October 2016, 1967 people were assessed and 1880 (95.6%) met eligibility criteria and chose to enroll. Demographic data were available on 1876 participants; their median age was 23 years, 671 (35.8%) had more than a secondary school education, and 435 (23.2%) identified as transgender women (Table 1).

**Table 1:** Baseline characteristics of participants in the test, treat, and prevent HIV program, Thailand, 2015–2016.

Characteristics	Total n = 1876 Number (%)	Men who have sex with men n = 1441 Number (%)	Transgender women n = 435 Number (%)	P-value*
**Recruitment method**				
Peer-driven intervention	443 (23.6)	316 (21.9)	127 (29.2)	
Walked in to clinic	1433 (76.4)	1125 (78.1)	308 (70.8)	0.002
**Site enrolled**				
Khon Kaen Hospital	333 (17.8)	243 (16.9)	90 (20.7)	
Lerdsin Hospital	167 (8.9)	144 (10.0)	23 (5.3)	
Srinagarind Hospital	393 (21.2)	300 (20.8)	98 (22.5)	
Thammasat Hospital	483 (25.8)	383 (26.6)	100 (23.0)	
Udon Thani Hospital	495 (26.4)	371 (25.8)	124 (28.5)	0.007
**How do you perceive yourself?**				
Heterosexual man	319 (17.0)	319 (22.1)	0 (0.0)	
Gay man	1031 (55.0)	1031 (71.6)	0 (0.0)	
Bisexual man	91 (4.9)	91 (6.3)	0 (0.0)	
Transgender woman	435 (23.2)	0 (0.0)	435 (100.0)	<0.0001
**Age at enrollment**				
Median age in years (Interquartile range)	23 (20–29)	23 (19–29)	24 (20–29)	0.30
18–21 years	778 (41.5)	621 (43.1)	157 (36.1)	
>21 years	1098 (58.5)	820 (56.9)	278 (63.9)	0.009
**Education**				
Primary (6 years) or less	175 (9.3)	128 (8.9)	47 (10.8)	
At least some secondary (7–12 years)	1030 (54.9)	753 (52.3)	277 (63.7)	
More than secondary	671 (35.8)	560 (38.9)	111 (25.5)	<0.0001
**Monthly income**				
<5000 baht (<US $145)	574 (30.6)	429 (29.8)	145 (33.3)	
5000–10,000 baht (US $146–280)	796 (42.4)	594 (41.2)	202 (46.4)	
>10,000 baht (>US $280)	506 (27.0)	418 (29.0)	88 (20.2)	0.001
**Current relationship status**				
Single	1200 (64.0)	896 (62.2)	304 (69.9)	
Have or live with male partner	601 (32.0)	472 (32.8)	129 (29.7)	
Have or live with female partner	75 (4.0)	73 (5.1)	2 (0.5)	<0.0001
**Age first had sex (data available on 1875 participants)**	**n = 1875**	**n = 1440**	**n = 435**	
Median age in years (Interquartile range)	17 (15–19)	17 (15–19)	16 (14–18)	
≤13 years	220 (11.7)	139 (9.7)	81 (18.6)	
14–16 years	634 (33.8)	458 (31.8)	176 (40.5)	
>16 years	1021 (54.5)	843 (58.5)	178 (40.9)	<0.0001
**Sexual activities in the past 6 months (data available on 1793 participants)**	**n = 1793**	**n = 1373**	**n = 420**	
Had sex with a man	1602 (89.6)	1190 (86.7)	416 (99.1)	<0.0001
Had sex with a woman	281 (15.7)	277 (20.2)	4 (1.0)	<0.0001
Had sex with a transgender woman	256 (14.3)	248 (18.1)	8 (1.9)	<0.0001
Had sex with an HIV-infected partner	86 (4.8)	74 (5.4)	12 (2.9)	0.03
100% condom use with HIV-infected partner (n = 86)	20 (23.3)	20 (27.0)	0 (0.0)	0.04
Had receptive anal intercourse	1186 (66.2)	806 (58.7)	380 (90.5)	<0.0001
100% condom use during receptive anal intercourse (n = 1186)	216 (18.2)	157 (19.5)	59 (15.5)	0.10
Had insertive anal intercourse	965 (53.8)	904 (65.8)	61 (14.5)	<0.0001
100% condom use during insertive anal intercourse (n = 965)	216 (22.4)	206 (22.8)	10 (16.4)	0.25
Had a steady sex partner	896 (50.0)	722 (52.6)	174 (41.4)	<0.0001
100% condom use with steady partner (n = 896)	143 (16.0)	127 (17.6)	16 (9.2)	0.007
Had casual sex partner/s	1233 (68.8)	912 (66.4)	321 (76.4)	0.0001
100% condom use with casual partner/s (n = 1233)	371 (30.1)	281 (30.8)	90 (28.0)	0.35
Had sex with sex worker/s	259 (14.5)	199 (14.5)	60 (14.3)	0.92
100% condom use with sex worker/s (n = 259)	88 (34.0)	71 (35.7)	17 (28.3)	0.29
Received money or gifts for sex	355 (19.8)	194 (14.1)	161 (38.3)	<0.0001
100% condom use with those who gave money or gifts (n = 355)	116 (32.7)	55 (28.4)	61 (37.9)	0.06
**Drug use activities in the past 6 months (data available on 1875 participants)**	**n = 1875**	**n = 1440**	**n = 435**	
Injected drugs	37 (2.0)	21 (1.5)	16 (3.7)	0.003
Drugs used (reported by >1% of participants)				
Alcohol	104 (5.6)	79 (5.5)	25 (5.8)	0.83
Methamphetamine	76 (4.1)	63 (4.4)	13 (3.0)	0.20
Poppers	36 (1.9)	36 (2.5)	0 (0.0)	0.0009
Marijuana	32 (1.7)	27 (1.9)	5 (1.2)	0.31
Viagra	30 (1.6)	29 (2.0)	1 (0.2)	0.01
**Baseline laboratory test results (data available on 1876 participants)**	**n = 1876**	**n = 1441**	**n = 435**	
First HIV test	1312 (69.9)	1029 (71.4)	283 (65.1)	0.01
HIV-infected	302 (16.1)	263 (18.3)	39 (9.0)	<0.0001
Median CD4 count (cells/mm^3^) (data available on 279 participants: MSM = 246, TG = 33)	312 (205–418)	310 (202–397)	323 (250–484)	0.25

MSM men who have sex with men, TG transgender women

*Chi-square test used for categorical variables and t-test used for continuous variables.

Among those who enrolled, 1602 (89.6%) reported having sex with a man in the previous 6 months, 281 (15.7%) had sex with a woman, and 256 (14.3%) with a transgender woman (Table 1). All participants reported having sex with a man or a transgender woman. Among the 1031 (55.0%) participants who identified as gay, 784 (76.0%) presented themselves as gay in public and 222 (21.5%) presented themselves as heterosexual; 420 (96.6%) of the 435 transgender women presented themselves as transgender women (data not shown). Among participants who identified as heterosexual men, 137 (47.2%) reported anal sex with a man in the previous 6 months and 180 (62.1%) reported anal sex with a transgender woman.

### Risk behavior

Participants reported first sex at a median age of 17 years; 139 (9.7%) of MSM and 81 (18.6%) of transgender women reported having sexual intercourse when they were 13 years old or younger (Table 1). A total of 1186 (66.2%) participants reported having receptive anal intercourse in the 6 months before enrollment, 965 (53.8%) had insertive anal intercourse, and 571 (31.8%) had both; 216 (18.2%) reported 100% condom use with receptive anal intercourse and 216 (22.4%) with insertive anal intercourse. Transgender women were more likely to report receptive anal intercourse than MSM (p<0.0001). A total of 259 (14.5%) participants reported sex with a sex worker in the previous 6 months and 355 (19.8%) reported receiving money or gifts for sex, including 161 (38.3%) of transgender women. Among those who had sex with sex workers, 88 (34.0%) reported 100% condom use and among those who received money or gifts for sex, 116 (32.7%) reported 100% condom use.

Drug use was modest with 37 (2.0%) participants reporting they had injected drugs in the previous 6 months and 188 (10.0%) reporting non-injection drug use: 104 (5.6%) used alcohol and 76 (4.1%) methamphetamines; 30 (1.6%) participants reported they had used medicine to enhance penile erection.

### Knowledge and attitudes about HIV, ART, and PrEP

Overall, 1764 (94.1%) participants reported that HIV could be transmitted by sexual intercourse and 1436 (76.6%) by sharing injection equipment (Table 2). A total of 1726 (92.1%) participants reported that condom use could reduce their risk of HIV infection. A smaller proportion reported that withdrawal before ejaculation (349 [18.6%]), cleaning their genital area after sex (343 [18.3%]), and choosing partners who looked healthy (195 [10.4%]) could reduce the risk of HIV transmission.

**Table 2:** Knowledge and attitudes of test, treat, and prevent HIV program participants about HIV infection, antiretrovirals, and pre-exposure prophylaxis, Thailand, 2015–2016.

Knowledge and attitudes	Total n = 1875 Number (%)	Men who have sex with men n = 1440 Number (%)	Transgender women n = 435 Number (%)	Chi-square p-value
**How can HIV be transmitted? Answered ‘yes’.**				
By vaginal or anal intercourse	1764 (94.1)	1366 (94.9)	398 (91.5)	0.009
By sharing injecting equipment (needles) with a PLHIV	1436 (76.6)	1128 (78.3)	308 (70.8)	0.001
From a mother who has HIV to her infant during pregnancy	952 (50.8)	768 (53.3)	184 (42.3)	<0.0001
By kissing	354 (18.9)	284 (19.7)	70 (16.1)	0.09
By taking care of a PLHIV	82 (4.4)	73 (5.1)	9 (2.1)	0.007
**Which of these methods can reduce your risk of HIV infection?**				
The correct use of condoms	1726 (92.1)	1328 (92.2)	398 (91.5)	0.62
Withdrawal before ejaculation	349 (18.6)	277 (19.2)	72 (16.6)	0.21
Cleaning genital areas after sexual intercourse	343 (18.3)	272 (18.9)	71 (16.3)	0.22
Choosing sex partners who look healthy	195 (10.4)	156 (10.8)	39 (9.0)	0.26
Being circumcised and only having sex with circumcised men	95 (5.1)	78 (5.4)	17 (3.9)	0.21
**Do you know anyone who is HIV-infected?**				
Yes	523 (27.9)	374 (26.0)	149 (34.3)	
No or don’t know	1352 (72.1)	1066 (74.0)	286 (65.8)	0.0007
**Have you ever received information about antiretroviral medicine (ART)?**				
Yes	669 (35.7)	519 (36.0)	150 (34.5)	
No	1206 (64.3)	921 (64.0)	285 (65.5)	0.55
**From what source did you receive information about ART? (data available on 669 participants)**	n = 669	n = 519	n = 150	
The internet	384 (57.4)	322 (62.0)	62 (41.3)	<0.0001
A hospital or medical clinic	359 (53.7)	272 (52.4)	87 (58.0)	0.23
Friends or relatives	220 (32.9)	169 (32.6)	51 (34.0)	0.74
Peer educators	205 (30.6)	143 (27.6)	62 (41.3)	0.001
Radio or television	134 (20.0)	107 (20.6)	27 (18.0)	0.48
Newspaper or magazine	134 (20.0)	113 (21.8)	21 (14.0)	0.04
**Who should start ART? (data available on 1875 participants)**	**n = 1875**	**n = 1440**	**n = 435**	
Everyone with HIV	1120 (59.7)	856 (59.4)	264 (60.7)	0.64
**If you received an HIV positive result today would you start ART?**				
Yes	1608 (85.8)	1231 (85.5)	377 (86.7)	
No or don’t know	267 (14.2)	209 (14.5)	58 (13.3)	0.54
**What are your concerns about ART?**				
Not concerned	461 (24.6)	327 (22.7)	134 (30.8)	0.0006
What my family will think if they find out I am taking ART	566 (30.2)	457 (31.7)	109 (25.1)	0.008
What my friends will think if they find out I am taking ART	507 (27.0)	411 (28.5)	96 (22.1)	0.008
Cost of medicines and treatment	692 (36.9)	556 (38.6)	136 (31.3)	0.005
Kidney problems	621 (33.1)	517 (35.9)	104 (23.9)	<0.0001
Liver problems	538 (28.7)	451 (31.3)	87 (20.0)	<0.0001
Rash	410 (21.9)	338 (23.5)	72 (16.6)	0.002
Anemia	213 (11.4)	172 (11.9)	41 (9.4)	0.15
Lipodystrophy	198 (10.6)	163 (11.3)	35 (8.1)	0.05
Taking medicine every day	642 (34.2)	520 (36.1)	122 (28.1)	0.002
**Does taking ART decrease the risk a person living with HIV will give HIV to a sexual partner?**				
Yes	1372 (73.2)	1057 (73.4)	315 (72.4)	
No	503 (26.8)	383 (26.6)	120 (27.6)	0.68
**Pre-exposure prophylaxis (PrEP) (data available on 360 participants)**	n = 360	n = 281	n = 79	
**Have you heard that taking ART can help prevent HIV infection?**				
Yes	207 (57.5)	160 (56.9)	47 (59.5)	
No or not sure	153 (42.5)	121 (43.1)	32 (40.5)	0.68
**If PrEP were available, would you use it?**				
Yes, definitely	235 (65.3)	173 (61.6)	62 (78.5)	
Maybe, probably not, or no	125 (34.7)	108 (38.4)	17 (21.5)	0.005
**How much would you be willing to pay per month?**				
Won’t pay	33 (9.2)	30 (10.7)	3 (3.8)	
<500 baht ($15 US)	148 (41.1)	105 (37.4)	43 (54.4)	
500–1000 baht ($15-$30 US)	127 (35.3)	104 (37.0)	23 (29.1)	
1001–3000 baht ($31-$88 US)	52 (14.4)	42 (14.9)	10 (12.7)	0.04
**What are important barriers to taking PrEP?**				
Cost	200 (55.6)	161 (57.3)	39 (49.4)	0.21
Side effects	222 (61.7)	182 (64.8)	40 (50.6)	0.02
Afraid others will think I have HIV	111 (30.8)	91 (32.4)	20 (25.3)	0.23
Afraid my family will find out I am taking PrEP	75 (20.8)	62 (22.1)	13 (16.5)	0.28
Afraid my sexual partner will find out I am taking PrEP	40 (11.1)	31 (11.0)	9 (11.4)	0.93
Afraid others will find out I am gay/a transgender woman	16 (4.4)	16 (5.7)	0 (0.0)	0.03

Although only 669 (35.7%) participants reported they had received information about ART, 1120 (59.7%) said that everyone with HIV should start ART, 1372 (73.2%) knew that PLHIV taking ART were less likely to transmit HIV to a sexual partner, and 1608 (85.8%) reported they would start ART if their HIV test result was positive. A total of 384 (57.4%) participants received information about ART from the internet and 359 (53.7%) from hospital or clinic staff. A total of 461 (24.6%) participants said they had no concerns about taking ART, 692 (36.9%) were concerned about cost, 642 (34.2%) about taking medicine daily, 621 (33.1%) about kidney problems, and 566 (30.2%) about what family members would think (Table 2).

Among the 360 HIV-uninfected participants at Lerdsin and Thammasat Hospitals who agreed to complete the PrEP questionnaire, 207 (57.5%) had heard taking ART could reduce the risk of HIV infection (Table 2). A total of 235 (65.3%) participants said they would definitely be willing to take PrEP and 148 (41.1%) would pay up to 500 baht ($15 US dollars) and 127 (35.3%) would pay 501 to 1000 baht ($15 to $30 US dollars) per month. Concern about PrEP side effects was reported as an important barrier by 222 (61.7%) participants, while 111 (30.8%) were concerned others would think they were HIV-infected.

### HIV testing results and ART use

All participants received their HIV result and, among the 1876 participants with baseline data, 1312 (69.9%) were first-time HIV testers and 302 (16.1%) had a positive HIV test result: 263 (18.3%) MSM and 39 (9.0%) transgender women (Table 1). A higher proportion of participants tested HIV-positive at Lerdsin (33.3%) and Udon Thani (23.8%) than at the other hospitals (range 8.7% to 12.8%). The median CD4 count of HIV-infected participants at diagnosis was 312 cells/mm^3^ and was similar in MSM and transgender women (p = 0.25).

In multivariable analysis, participants who walked in to the clinic were more likely to be HIV-infected (18.4%) than those recruited by peers (8.6%) (odds ratio [OR] 2.5, 95% confidence interval [CI] 1.6–3.7), MSM were more likely to be HIV-infected (18.3%) than transgender women (9.0%) (OR 2.6, 95% CI 1.7–4.0), participants who earned more than 10,000 baht per month were more likely to be HIV-infected than those who earned less than 5000 baht (OR 1.6, 95% CI 1.1–2.5), and participants who reported receptive anal intercourse during the 6 months before enrollment were more likely to be HIV-infected than those who did not (OR 2.7, 95% CI 2.0–3.8). Participants who reported that they were 16 years or older when they first had sexual intercourse were less likely to be HIV-infected than participants who had sexual intercourse when they were 13 years old or younger (OR 0.6, 95% CI 0.4–1.0) and those who reported having sex with sex workers during the 6 months before enrollment were less likely to be HIV-infected at baseline (OR 0.5, 95% CI 0.3–0.7) than those who did not report sex with sex workers (Table 3).

**Table 3:** Results of logistic regression analysis to evaluate characteristics of participants in the test, treat, and prevent HIV program associated with prevalent HIV infection, Thailand, 2015–2016.

Characteristics (data available on 1880 participants)	HIV-infected at baseline	Bivariable analysis		Multivariable analysis	
		Odds Ratio (95% CI)	P-value	Odds Ratio (95% CI)	P-value
**Site enrolled**^**a**^					
Khon Kaen Hospital (n = 334)	29 (8.7)	1.0		1.0	
Lerdsin Hospital (n = 168)	56 (33.3)	5.3 (3.2–8.7)	<0.0001	2.4 (1.3–4.2)	0.003
Srinagarind Hospital (n = 400)	38 (9.5)	1.1 (0.7–1.8)	0.70	0.7 (0.4–1.2)	0.21
Thammasat Hospital (n = 483)	62 (12.8)	1.5 (1.0–2.5)	0.07	0.8 (0.5–1.4)	0.48
Udon Thani Hospital (n = 495)	118 (23.8)	3.3 (2.1–5.1)	<0.0001	2.5 (1.5–3.9)	0.0002
**Recruitment method**					
Peer-driven intervention (n = 443)	38 (8.6)	1.0		1.0	
Walked in to clinic (n = 1437)	265 (18.4)	2.9 (2.0–4.2)	<0.0001	2.5 (1.6–3.7)	<0.0001
**Age at enrollment**					
18–21 years (n = 782)	91 (11.6)	1.0		1.0	
≥22 years (n = 1098)	212 (19.3)	1.6 (1.2–2.2)	0.0004	1.8 (1.3–2.5)	0.0006
**Self-identified (data available on 1876 participants)**					
Transgender women (n = 435)	39 (9.0)	1.0		1.0	
Man who has sex with men (n = 1441)	263 (18.3)	2.2 (1.5–3.2)	<0.0001	2.6 (1.7–4.0)	<0.0001
**Education**					
Secondary or less (n = 1205)	168 (13.9)	1.0		1.0	
More than secondary (n = 671)	134 (20.0)	1.3 (1.0–1.7)	0.06	0.9 (0.6–1.2)	0.39
**Income per month**					
<5000 baht (n = 574)	69 (12.0)	1.0		1.0	
5000–10,000 (n = 796)	110 (13.8)	1.2 (0.8–1.6)	0.31	1.0 (0.7–1.4)	0.93
>10,000 (n = 506)	123 (24.3)	1.9 (1.3–2.7)	0.0003	1.6 (1.1–2.5)	0.02
**Ever had an HIV test**					
Yes (n = 564)	59 (10.5)	1.0		1.0	
No (n = 1312)	243 (18.5)	2.5 (1.8–3.5)	<0.0001	3.0 (2.1–4.3)	<0.0001
**Age at first sex (data available on 1875 participants)**					
≤13 years (n = 220)	42 (19.1)	1.0		1.0	
14–16 years (n = 634)	88 (13.9)	0.6 (0.4–0.9)	0.02	0.7 (0.4–1.1)	0.09
>16 years (n = 1021)	171 (16.8)	0.7 (0.5–1.1)	0.11	0.6 (0.4–1.0)	0.04
**In the 6 months before enrollment**					
**Sexual activity (data available on 1793 participants)**					
**Had sex with HIV-infected partner or partner of unknown HIV status**					
No (n = 1707)	276 (16.2)	1.0			
Yes (n = 86)	18 (20.9)	1.2 (0.7–2.1)	0.54	Not included	
**Had receptive anal intercourse**					
No (n = 607)	60 (9.9)	1.0		1.0	
Yes (n = 1186)	234 (19.7)	2.1 (1.5–2.9)	<0.0001	2.7 (2.0–3.8)	<0.0001
**Had insertive anal intercourse**					
No (n = 828)	122 (14.7)	1.0		1.0	
Yes (n = 965)	172 (17.8)	1.3 (1.0–1.6)	0.08	1.0 (0.8–1.4)	0.85
**Had sex with a steady partner**					
No (n = 897)	147 (16.4)	1.0			
Yes (n = 896)	147 (16.4)	0.9 (0.7–1.2)	0.67	Not included	
**Had sex with a casual partner/s**					
No (n = 560)	97 (17.3)	1.0			
Yes (n = 1233)	197 (16.0)	1.0 (0.7–1.3)	0.86	Not included	
**Had sex with sex worker/s**					
No (n = 1534)	268 (17.5)	1.0		1.0	
Yes (n = 259)	26 (10.0)	0.6 (0.4–0.9)	0.02	0.5 (0.3–0.7)	0.001
**Was paid or received gifts for sex**					
No (n = 1438)	246 (17.1)	1.0			
Yes (n = 355)	48 (13.5)	0.8 (0.6–1.1)	0.21	Not included	
**Drug use (data available on 1875 participants)**					
**Injected drugs**					
No (n = 1838)	294 (16.0)	1.0			
Yes (n = 37)	7 (18.9)	1.2 (0.5–2.7)	0.73	Not included	
**Used alcohol**					
No (n = 1771)	286 (16.2)	1.0			
Yes (n = 104)	15 (14.4)	0.9 (0.5–1.7)	0.79	Not included	
**Used methamphetamine**					
No (n = 1799)	280 (15.6)	1.0		1.0	
Yes (n = 76)	21 (27.6)	1.9 (1.1–3.2)	0.02	1.4 (0.8–2.7)	0.25
**Used poppers**					
No (n = 1839)	287 (15.6)	1.0		1.0	
Yes (n = 36)	14 (38.9)	2.4 (1.2–4.8)	0.02	1.6 (0.7–3.7)	0.25
**Used marijuana**					
No (n = 1843)	298 (16.2)	1.0			
Yes (n = 32)	3 (9.4)	0.6 (0.2–2.0)	0.40	Not included	
**Used Viagra**					
No (n = 1845)	292 (15.8)	1.0			
Yes (n = 30)	9 (30.0)	1.4 (0.6–3.2)	0.40	Not included	

CI, confidence interval

^a^We included the site of enrollment in all models to control for clustering by site.

Among the 302 participants with a positive HIV test result and demographic data, 269 (89.1%) had reported they would start ART if they tested HIV-positive and, of these, 235 (87.4%) started ART (data not shown). Overall, 260 (86.1%) of those who tested HIV-positive started ART, ranging from 54 (98.2%) at Lerdsin and 112 (94.9%) at Udon to 43 (69.4%) at Thammasat; 177 (58.6%) started within 2 weeks of the positive HIV test. In multivariable analysis, participants who earned more than 10,000 baht ($285 US) were more likely to start ART (93.5%) than participants who earned less than 5000 baht ($143 US) (79.7%) (OR 3.4, 95% CI 1.1–10.4) (Table 4).

**Table 4:** Results of logistic regression analysis evaluating characteristics of participants in the test, treat, and prevent HIV program with prevalent HIV infection who chose to start antiretroviral therapy (ART), Thailand, 2015–2016.

Characteristics (data available on 302 participants)	Started ART	Bivariable analysis		Multivariable analysis	
		Odds Ratio (95% CI)	P-value	Odds Ratio (95% CI)	P-value
**Site enrolled**^**a**^					
Khon Kaen Hospital (n = 29)	22 (75.9)	1.0		1.0	
Lerdsin Hospital (n = 55)	54 (98.2)	17.2 (2.0–148.0)	0.01	13.9 (1.5–129.8)	0.02
Srinagarind Hospital (n = 38)	29 (76.3)	1.0 (0.3–3.2)	0.97	1.3 (0.4–4.6)	0.64
Thammasat Hospital (n = 62)	43 (69.4)	0.7 (0.3–2.0)	0.52	1.0 (0.3–3.1)	0.99
Udon Thani Hospital (n = 118)	112 (94.9)	5.9 (1.8–19.4)	0.003	7.6 (2.1–27.3)	0.002
**Recruitment method**					
Peer-driven intervention (n = 38)	31 (81.6)	1.0			
Walked in to clinic (n = 264)	229 (86.7)	2.1 (0.7–7.0)	0.20	Not included	
**Self-identified as**					
Transgender woman (n = 39)	31 (79.5)	1.0			
Man who has sex with men (n = 263)	229 (87.1)	1.3 (0.5–3.2)	0.60	Not included	
**Age at enrollment**					
18–21 years (n = 90)	74 (82.2)	1.0			
≥22 years (n = 212)	186 (87.7)	1.4 (0.7–2.8)	0.41	Not included	
**Education**					
Secondary or less (n = 168)	138 (82.1)	1.0			
More than secondary (n = 134)	122 (91.0)	1.5 (0.7–3.3)	0.26	Not included	
**Income per month**					
<5000 baht (n = 69)	55 (79.7)	1.0		1.0	
5000–10,000 baht (n = 110)	90 (81.8)	1.8 (0.8–4.3)	0.18	1.2 (0.5–3.1)	0.68
>10,000 bath (n = 123)	115 (93.5)	4.6 (1.7–12.8)	0.003	3.4 (1.1–10.4)	0.03
**Know a person living with HIV**					
No (n = 206)	173 (84.0)	1.0		1.0	
Yes (n = 96)	87 (90.6)	2.0 (0.9–4.5)	0.10	2.0 (0.8–5.2)	0.14
**Age at first sex (data available on 301 participants)**					
≤13 years (n = 42)	31 (73.8)	1.0		1.0	
14–16 years (n = 88)	81 (92.1)	3.3 (1.1–9.8)	0.03	3.4 (1.0–11.4)	0.05
>16 years (n = 171)	147 (86.0)	1.7 (0.7–4.1)	0.23	1.5 (0.6–3.9)	0.42
**In the 6 months before enrollment**					
**Sexual activity (data available on 294 participants)**					
**Had receptive anal intercourse**					
No (n = 60)	51 (85.0)	1.0			
Yes (n = 234)	203 (86.8)	1.3 (0.5–3.0)	0.59	Not included	
**Had insertive anal intercourse**					
No (n = 122)	105 (86.1)	1.0			
Yes (n = 172)	149 (86.6)	0.9 (0.5–1.9)	0.89	Not included	
**Had sex with a casual partner/s**					
No (n = 97)	83 (85.6)	1.0			
Yes (n = 197)	171 (86.8)	1.2 (0.6–2.5)	0.64	Not included	
**Had sex with sex worker/s**					
No (n = 268)	235 (87.7)	1.0		1.0	
Yes (n = 26)	19 (73.1)	0.4 (0.2–1.2)	0.12	0.3 (0.09–1.2)	0.09
**Was paid or received gifts for sex**					
No (n = 246)	217 (88.2)	1.0		1.0	
Yes (n = 48)	37 (77.1)	0.5 (0.2–1.1)	0.08	0.9 (0.3–2.7)	0.92
**Drug use (data available on 301 participants)**					
**Injected drugs**					
No (n = 294)	254 (86.4)	1.0		1.0	
Yes (n = 7)	5 (71.4)	0.2 (0.03–1.5)	0.11	0.2 (0.02–1.5)	0.12
**Used drugs (not injected)**^**b**^					
No (n = 265)	230 (86.8)	1.0			
Yes (n = 36)	29 (80.6)	0.5 (0.2–1.4)	0.17	Not included	
**Received information about antiretroviral medicines in the past (data available on 302 participants)**					
No (n = 204)	171 (83.8)	1.0			
Yes (n = 98)	89 (90.8)	1.8 (0.9–4.1)	0.17	Not included	
**Do you have concerns about antiretroviral medicines**					
No (n = 50)	40 (80.0)	1.0			
Yes (n = 252)	220 (87.3)	1.8 (0.8–4.3)	0.17	Not included	
**Concerned about anemia**					
No (n = 270)	233 (86.3)	1.0			
Yes (n = 32)	27 (84.4)	1.0 (0.3–2.8)	0.94	Not included	
**Concerned about cost of medicine**					
No (n = 190)	164 (85.7)	1.0			
Yes (n = 112)	96 (85.7)	1.2 (0.6–2.4)	0.70	Not included	
**Concerned about kidney problems**					
No (n = 197)	166 (84.3)	1.0			
Yes (n = 105)	94 (89.5)	1.5 (0.7–3.4)	0.27	Not included	
**Concerned about lipodystrophy**					
No (n = 251)	215 (85.7)	1.0			
Yes (n = 51)	45 (88.2)	1.3 (0.5–3.5)	0.58	Not included	
**Concerned about liver problems**					
No (n = 213)	181 (85.0)	1.0			
Yes (n = 89)	79 (88.8)	1.3 (0.6–3.0)	0.47	Not included	
**Concerned about rash**					
No (n = 221)	186 (84.2)	1.0			
Yes (n = 81)	74 (91.4)	1.5 (0.6–3.6)	0.40	Not included	
**Concerned about taking medicine every day**					
No (n = 178)	153 (86.0)	1.0			
Yes (n = 124)	107 (86.3)	0.9 (0.4–1.8)	0.69	Not included	
**Concerned about what my family with think**					
No (n = 173)	149 (86.1)	1.0			
Yes (n = 129)	111 (86.1)	0.9 (0.4–1.8)	0.70	Not included	
**Concerned about what my friends with think**					
No (n = 192)	165 (85.9)	1.0			
Yes (n = 110)	95 (86.4)	1.0 (0.5–2.1)	0.98	Not included	
**Know that taking antiretroviral medicine will decrease the risk a person who is infected will give HIV to a sexual partner**					
No (n = 102)	89 (87.3)	1.0			
Yes (n = 200)	171 (85.5)	0.7 (0.3–1.6)	0.43	Not included	

CI, confidence interval.

^a^We included the site of enrollment in all models to control for clustering by site.

^b^For example: alcohol, barbiturates, cocaine, heroin, ketamine, marijuana, methamphetamine, 3,4-methylenedioxy-methamphetamine (ecstasy), nitrites (poppers).

### PrEP

Among the 534 participants at Thammasat and Lerdsin Hospitals who tested HIV-negative, 367 (68.70%) agreed to hear more about PrEP and 167 (45.5%) of these participants started PrEP: 84 (29.4%) at Thammasat and 84 (98.8%) at Lerdsin (Table 5).

**Table 5:** Results of logistic regression analysis evaluating characteristics of HIV-uninfected participants in the test, treat, and prevent HIV program at Lerdsin and Thammasat University Hospitals who chose to start HIV pre-exposure prophylaxis (PrEP), Thailand, 2015–2016.

Characteristics (data available on 367 participants)	Started PrEP	Bivariable analysis		Multivariable analysis	
		Odds Ratio (95% CI)	P-value	Odds Ratio (95% CI)	P-value
**Site enrolled**^**a**^					
Thammasat Hospital (n = 282)	83 (29.4)	1.0		1.0	
Lerdsin Hospital (n = 85)	84 (98.8)	201.4 (27.6->1000)	<0.0001	274.2 (34.4->1000)	<0.0001
**Recruitment method**					
Walked in to clinic (n = 350)	151 (43.1)	1.0		1.0	
Peer-driven intervention (n = 17)	16 (94.1)	7.7 (0.8–73.5)	0.08	6.9 (0.6–75.6)	0.11
**Self-identified as**					
Man who has sex with men (n = 287)	125 (43.6)	1.0		1.0	
Transgender woman (n = 80)	42 (52.5)	1.6 (0.9–3.0)	0.10	1.3 (0.6–3.2)	0.50
**Age at enrollment**					
18–21 years (n = 115)	50 (43.5)	1.0		1.0	
≥22 years (n = 252)	117 (46.4)	0.6 (0.4–1.1)	0.08	0.5 (0.3–1.0)	0.07
**Education**					
Secondary or less (n = 215)	89 (41.4)	1.0			
More than secondary (n = 152)	78 (51.3)	0.9 (0.5–1.5)	0.57	Not included	
**Income (Thai baht)**					
<5000 baht (n = 59)	25 (42.4)	1.0			
5000–10,000 baht (n = 151)	55 (36.4)	0.8 (0.4–1.7)	0.60		
>10,000 baht (n = 157)	87 (55.4)	0.7 (0.4–1.5)	0.42	Not included	
**Know a person living with HIV**					
No (n = 276)	115 (41.7)	1.0		1.0	
Yes (n = 91)	52 (57.1)	1.7 (0.9–3.1)	0.07	1.8 (0.9–3.7)	0.10
**Age at first sex**					
≤13 years (n = 29)	9 (31.0)	1.0			
14–16 years (n = 108)	43 (39.8)	1.8 (0.6–5.7)	0.31	2.2 (0.6–8.0)	0.24
>16 years (n = 230)	115 (50.0)	2.3 (0.8–6.9)	0.13	3.9 (1.1–13.4)	0.03
**In the 6 months before enrollment**					
**Sexual activity (data available on 348 participants)**					
**Had sex with HIV-infected partner or partner of unknown HIV status**					
No (n = 323)	139 (43.0)	1.0		1.0	
Yes (n = 25)	22 (88.0)	5.8 (1.5–22.9)	0.01	12.4 (2.6–59.7)	0.002
**Had receptive anal intercourse**					
No (n = 133)	47 (35.3)	1.0		1.0	
Yes (n = 215)	114 (53.0)	2.9 (1.6–5.2)	0.0004	2.4 (1.2–4.8)	0.02
**Had insertive anal intercourse**					
No (n = 150)	76 (50.7)	1.0		1.0	
Yes (n = 198)	85 (42.9)	0.6 (0.4–1.1)	0.10	0.7 (0.4–1.5)	0.40
**Had sex with a steady partner**					
No (n = 182)	73 (40.1)	1.0			
Yes (n = 166)	88 (53.0)	1.3 (0.8–2.2)	0.31	Not included	
**Had sex with a casual partner/s**					
No (n = 98)	49 (50.0)	1.0			
Yes (n = 250)	112 (44.8)	1.2 (0.7–2.2)	0.52	Not included	
**Had sex with sex worker/s**					
No (n = 299)	141 (47.2)	1.0			
Yes (n = 49)	20 (40.8)	0.9 (0.5–1.9)	0.87	Not included	
**Was paid or received gifts for sex**					
No (n = 290)	137 (47.2)	1.0			
Yes (n = 58)	24 (41.4)	0.6 (0.3–1.2)	0.16	Not included	
**Drug use (data available on 367 participants)**					
**Injected drugs**					
No (n = 362)	163 (45.0)	1.0		1.0	
Yes (n = 5)	4 (80.0)	7.4 (0.8–72.5)	0.08	5.3 (0.4–71.8)	0.21
**Used drugs (not injected)**					
No (n = 327)	145 (44.3)	1.0			
Yes (n = 40)	22 (55.0)	1.4 (0.6–3.2)	0.41	Not included	
**Knowledge and attitudes (data available on 360 participants)**					
**Know ART (PrEP) can prevent HIV infection**					
No (n = 153)	60 (39.2)	1.0		1.0	
Yes (n = 207)	105 (50.7)	1.9 (1.1–3.2)	0.02	0.7 (0.3–1.4)	0.32
**Know of PrEP from internet**					
No (n = 276)	127 (46.0)	1.0			
Yes (n = 84)	38 (45.2)	1.1 (0.6–1.9)	0.85	Not included	
**Know of PrEP from hospital or clinic**					
No (n = 202)	67 (33.2)	1.0		1.0	
Yes (n = 158)	98 (62.0)	3.9 (2.3–6.8)	<0.0001	5.2 (2.5–10.8)	<0.0001
**Know of PrEP from radio or television**					
No (n = 352)	163 (46.3)	1.0			
Yes (n = 8)	2 (25.0)	0.4 (0.05–3.2)	0.39	Not included	
**Know of PrEP from newspaper or magazine**					
No (n = 337)	153 (45.4)	1.0		1.0	
Yes (n = 23)	12 (52.2)	2.3 (0.9–5.7)	0.07	4.8 (1.6–14.6)	0.006
**Know of PrEP from friends or relatives**					
No (n = 304)	143 (47.0)	1.0			
Yes (n = 56)	22 (39.3)	0.7 (0.3–1.4)	0.30	Not included	
**Think cost is an important barrier to PrEP use**					
No (n = 160)	80 (50.0)	1.0			
Yes (n = 200)	85 (42.5)	1.0 (0.6–1.7)	0.99	Not included	
**Think side effects are an important barrier to PrEP use**					
No (n = 138)	61 (44.2)	1.0			
Yes (n = 222)	104 (46.9)	0.9 (0.5–1.5)	0.59	Not included	

CI, confidence interval.

^a^We included the site of enrollment in all models to control for clustering by site.

Among the 360 participants who completed the PrEP attitudes questionnaire, 235 (65.3%) reported they would definitely use PrEP if it was available (Table 2); of these, 137 (58.3%) started PrEP. Of the 27 participants who said they would definitely or probably not take PrEP, 4 (14.8%) started PrEP. In multivariable analysis, participants who reported that they had sex with an HIV-infected partner or partner of unknown HIV status (OR 12.4, 95% CI 2.6–59.7), receptive anal intercourse (OR 2.4, 95% CI 1.2–4.8), or received information about PrEP from a hospital or clinic (OR 5.2, 95% CI 2.5–10.8) or a newspaper or magazine (4.8, 95% CI 1.6–14.6) were more likely to start PrEP than participants who did not report these behaviors or experiences (Table 5).

### Recruitment method

The number of participants recruited by peers ranged from 14 (2.9%) at Thammasat to 182 (54.5%) at Khon Kaen Hospital (data not shown). Participants recruited by peers were more likely to identify as a transgender women (Table 1), were younger (peer-recruited, mean age 23 years; walk-in, 26 years; p<0.0001, data not shown), and less likely to be HIV-infected (8.6% HIV-positive) than participants who walked into the clinics (18.4% HIV-positive; p<0.0001, data not shown). Among those who tested HIV-positive, peer-recruited participants had a higher mean CD4 count (388 cells/mm^3^) than participants who walked into the clinics (318 cells/mm^3^; p = 0.04, data not shown).

## Discussion

We worked with health care workers and peer educators to implement a Test, Treat, and Prevent HIV Program for MSM and transgender women at five hospital-based outpatient clinics in four provinces of Thailand offering rapid HIV testing, immediate access to ART, and, at two clinics, PrEP. Overall, 70% of MSM and transgender women who participated in the program were having an HIV test for the first time, highlighting the need to expand access to HIV testing. Among those who tested HIV-positive, 86.1% started ART, 58.6% started within two weeks and, among those who tested HIV-negative and were eligible, 45.5% started PrEP. ART uptake was higher than the estimated 68% of PLHIV on ART nationally [19], but lower than the 90% goal of UNAIDS [20] and the Thailand Ministry of Public Health [11]. At Lerdsin and Udon Thani more than 90% of HIV-positive participants started ART. These are large public hospitals with dedicated staff providing HIV services in one clinic site. The ability to access all services in one site may streamline activities and improve uptake of ART.

A higher proportion of participants started PrEP at Lerdsin than Thammasat. This may be because a higher proportion of participants at Lerdsin reported having sex with an HIV-infected partner or partner of unknown HIV status and were more likely to have heard about PrEP from hospital or clinic staff than participants at Thammasat. In addition, Lerdsin has a dedicated HIV clinic providing HIV services in one clinic, while at Thammasat Hospital these services are provided in clinics in different parts of the hospital. Stigma related to HIV infection remains an important problem, 30% of participants reported they were reluctant to use PrEP because they were concerned people would think they were HIV-infected. Participants who reported sex with an HIV-infected partner or receptive anal sex were more likely to start PrEP than participants who did not report these risk behaviors, suggesting that participants decided to take PrEP based on their self-determined risk of HIV infection. This is consistent with data from a PrEP study among PWID in Bangkok that found PWID who reported recent drug injection were more likely to start and adhere to PrEP than participants who did not report injection [21]. Participants who received information about PrEP from a hospital or clinic were more likely to start PrEP than those who did not, highlighting the impact health care staff can have on patient’s health-related choices.

Peer recruitment was most successful in Khon Kaen where there is a strong MSM network engaged in community and health activities. Participants recruited by peers were less likely to be HIV-infected and, among those who were infected, had higher CD4 counts than those who walked in to the clinics, suggesting that peers were able to reach people before they became infected, providing an opportunity to promote HIV prevention services including PrEP, and to expand the use of ART by those early in HIV illness. Peers recruited a higher proportion of transgender women (29%) than MSM (22%). Peer recruitment strategies may be useful tools for reaching transgender women.

HIV prevalence was higher among participants at Lerdsin and Udon Thani Hospitals than the other hospitals. Lerdsin is in central Bangkok close to entertainment venues that cater to MSM and the MOPH Sexually Transmitted Diseases Clinic. The HIV prevalence among people testing at Lerdsin may reflect the high prevalence and incidence of HIV among MSM in Bangkok [22, 23] and referral patterns. Udon Thani Hospital is located in northeastern Thailand and has a busy one-stop HIV testing and care clinic. Recruitment activities may have identified a population of MSM and transgender women at high risk of HIV infection. In addition, HIV prevalence surveys among MSM in Udon Thani Province in 2010–2012 found HIV prevalence of 6% to 7% [24, 25].

Although all participants reported condomless anal sex with a man or transgender woman, participants expressed diverse sexual identities. A substantial proportion (17%) described themselves as heterosexual and 22% of those who identified as gay, presented themselves as heterosexual in public, suggesting that stigma and discrimination continue to influence behavior. MSM and transgender women reported significantly different education levels, incomes, sexual activity, condom use behaviors, and HIV prevalence highlighting the importance of evaluating MSM and transgender women separately [26–29]. HIV prevalence among transgender women in the study (9.0%) is lower than reported in studies conducted in other low- and middle-income countries [29]. This study was not designed to provide an accurate estimate of HIV prevalence among transgender women in Thailand and more work needs to be done to address the burden of HIV among these women.

Thailand implemented nationwide HIV education efforts and a 100% condom use campaign in the late 1980s targeting sex workers and their partners which contributed to the control of an expanding generalized HIV epidemic [30, 31]. Despite this historic success, only 18% of MSM and transgender women in this project reported 100% condom use with receptive anal intercourse and only a third reported 100% condom use when paid for sex or when having sex with sex workers. Given the high HIV transmission probability of anal sex [32] and the high HIV prevalence among MSM in Thailand [1] the lack of consistent condom use suggests that condom use messages need to be developed for MSM and transgender women and appropriately targeted to these populations and a combination of prevention strategies including PrEP will be required to stem the ongoing epidemic. Almost 70% of MSM and transgender women, all of whom reported condomless anal sex in the previous 6 months, were testing for HIV for the first time. There is an urgent need to expand access to HIV testing among people at high risk of HIV infection. Participants who reported that they were 16 years or older when they first had sexual intercourse were less likely to be HIV-infected at enrollment than participants who reported having sexual intercourse when they were 13 years old or younger, demonstrating the importance of introducing risk reduction information to adolescents and young adults.

This study has several limitations. We collected data as a Test, Treat, and Prevent HIV program was implemented; there was no control group to compare results. Only two hospitals provided PrEP; thus, it is not clear if factors were associated with PrEP uptake or with the sites. The aim of this project was to implement a sustainable ART treatment and HIV PrEP program in public and university hospitals; we had to strictly limit data collection to essential elements focused on ART and PrEP uptake. Participants may have under-reported stigmatized behaviors [33].

## Conclusions

Results of this Test, Treat, and Prevent HIV Program suggest that compensated peer-driven recruitment can reach MSM and transgender women at high risk of HIV infection before they become infected or early in their HIV illness. We found that HIV-infected MSM and transgender women were willing to start ART immediately and, if HIV-uninfected, use PrEP, supporting government efforts to implement an HIV Test and Start model and PrEP [34]. The high HIV prevalence among MSM and transgender women highlights the urgent need to expand access to HIV testing, ART, and HIV prevention tools including PrEP and provide these life-saving services in settings that support people to engage and continue in care.

## Supporting information

S1 File Test, treat, and prevent HIV study data set. (XLSX)

S2 File Test, treat, and prevent study data dictionary. (XLSX)

S3 File Test, treat, and prevent study questionnaires. (ZIP)
