# Supplementary material for: Implementation of a Test, Treat, and Prevent HIV program among men who have sex with men and transgender women in Thailand, 2015-2016
Source: PLoS One. 2018 Jul 25;13(7):e0201171. doi: 10.1371/journal.pone.0201171 (PMC6059477; doi:10.1371/journal.pone.0201171)
Supplement: S3 File — (ZIP) [file pone.0201171.s003.zip › 2014 07 30 KPIS Risk Eng.docx]

**Behavioral questionnaire** (**approximately 5 minutes to complete)**

**VISIT: M0** □**M06** □**M12** □**M18**

- This questionnaire aims to collect information from participants in the study “Evaluation of the feasibility of a facility-based Test, Treat, and Prevent HIV program among men who have sex with men and transgender women in Thailand (Thai Facility-based MSM/TG Test, Treat, and Prevent HIV Study)”. Your responses will help us understand more about people’s attitudes towards taking anti-retroviral therapy regardless of CD4 level.
- You have the right to answer or not to answer any question. Answering or not answering will not have an negative impact on you. Your answers will help us understand your situation better.
- Your answers are confidential and will only be used for this study.
- Some questions may make you uncomfortable or embarrassed, we apologize for this.

**Please put an “X” in the box that best describes your situation**

**Questions 1 – 3 to be answered at the M0 visit only**

1. How old were you when you first had sexual intercourse?

_____ years old

1. In your life, who have you had sex with? (check all that apply)

□1 Women

□2 Men

□3 Transgender women

□4 I don’t want to answer

1. Are you circumcised?

□1 Yes

□1a. I was circumcised when I was _____ years old

□1b. I don’t know how old I was when I was circumcised

□2 No

□3 I don’t want to answer

**Visits other than M0, start at question # 4**

1. In the past 6 months, did you do anything (risk behaviors) that put you at risk of HIV infection?

□1 No, I did not do anything that put me at risk of HIV

□2 Yes, I did some things that put me at a low risk of HIV

□3 Yes, I did some things that put me at a moderate risk of HIV

□4 Yes, I did some things that put me at a high risk of HIV

1. In the past 6 months, did you have sex with anyone?

□1 No

□2 Yes

□3 I don’t want to answer

5.1 Who did you have sex with? (check all that apply)

□1 I had sex with male sex partners; #................ persons

□2 I had sex with female sex partners; #.................. persons

□3 I had sex with transgender sex partners; # ………………… persons

5.2 Where did you meet this/these sexual partner/s? (check all that apply)

□1 Restaurant

□2 Spa

□3 Sauna

□4 Fitness

□5 Pub / Bar / Disco

□6 Malls

□7 Website / Internet / Computer Applications

□8 None of the above

1. In the past 6 months, did you have sex with a partner who was HIV-infected?

□1 No

□2 Yes

□3 I don’t want to answer

□4 I don’t know if the partner is HIV-infected or not

6.1 Did you use a condom with this HIV-infected partner?

□1 No, I did not use condoms

□2 Yes, sometimes

□3 Yes, every time

□4 I don’t want to answer

1. In the past 6 months, did you have receptive anal intercourse?

□1 No

□2 Yes

□3 I don’t want to answer

7.1 Did your partner/s use condoms when you had receptive anal intercourse?

□1 No, they did not use condoms

□2 Yes, sometimes

□3 Yes, every time

□4 I don’t want to answer

1. In the past 6 months, did you have a steady sex partner?

□1 No

□2 Yes

□3 I don’t want to answer

8.1 Did you use condoms with your steady partner(s)?

□1 No, I did not use condoms

□2 Yes, sometimes

□3 Yes, every time

□4 I don’t want to answer

1. In the past 6 months, did you have a casual sex partner/s?

□1 No

□2 Yes

□3 I don’t want to answer

9.1Did you use condoms with your casual partner(s)?

□1 No, I did not use condoms

□2 Yes, sometimes

□3 Yes, every time

□4 I don’t want to answer

1. In the past 6 months, did you have sex with sex worker/s?

□1 No

□2 Yes

□3 I don’t want to answer

10.1 Did you use condoms with these sex workers?

□1 No, I did not use condoms

□2 Yes, sometimes

□3 Yes, every time

□4 I don’t want to answer

1. In the past 6 months, did you have sex with partner/s who paid you or gave you gifts?

□1 No

□2 Yes

□3 I don’t want to answer

11.1 Did you use condoms with these sex partners who paid you or gave you gifts?

□1 No, I did not use condoms

□2 Yes, sometimes

□3 Yes, every time

□4 I don’t want to answer

1. In the past 6 months, did you have sex with anyone who injects drugs?

□1 No

□2 Yes

□3 I don’t want to answer

12.1 Did you use condoms with these sex partners who inject drugs?

□1 No, I did not use condoms

□2 Yes, sometimes

□3 Yes, every time

□4 I don’t want to answer

1. In the past 6 months, have you injected drugs?

□1 No

□2 Yes

□3 I don’t want to answer

13.1 What drug/s did you inject? (check all that apply)

□1 Heroin

□2 Midazolam

□3 Methamphetamine

□4 Others, or not sure of the name of the drugs that I used

□5 I don’t want to answer

- 1. Did you share needles or injecting equipment with others?

□1 No

□2 Yes

□3 I don’t want to answer

1. In the past 6 months, did you use drugs (swallowed, inhaled, smoked, etc.) that you did not inject?

□1 No

□2 Yes

□3 I don’t want to answer

14.1 What drugs did you swallow, inhale, smoke, etc.? (check all that apply)

□1 Alcohol

□2 Methamphetamine

□3 Ecstasy

□4 Ketamine

□5 Poppers

□6 Heroin

□7 Cocaine

□8 Marijuana

□9 LSD

□10 Barbiturates

□11 Viagra

□12 Others /or not sure of the name of the drugs that I used

□13 I don’t want to answer

1. For what purpose did you use drugs?

□1 For sexual stimulation, before or during sex

□2 For long lasting erection

□3 To forget things that I don’t want to remember

□4 Curiosity or because friends persuaded me

□5 I am addicted

□6 I don’t want to answer

1. In the past 6 months, did you have any symptoms, or receive a diagnosis of a sexually transmitted disease such as gonorrhea, chlamydia, herpes, or syphilis?

□1 No

□2 Yes

□3 Not sure

□4 I don’t want to answer
