# Supplementary material for: Implementation of a Test, Treat, and Prevent HIV program among men who have sex with men and transgender women in Thailand, 2015-2016
Source: PLoS One. 2018 Jul 25;13(7):e0201171. doi: 10.1371/journal.pone.0201171 (PMC6059477; doi:10.1371/journal.pone.0201171)
Supplement: S3 File — (ZIP) [file pone.0201171.s003.zip › 2014.07.14 KPIS Demo Eng.docx]

**Demographic questionnaire**

(Approximately 5 minutes to complete)

**VISIT: Baseline**

- This questionnaire aims to collect information from participants in the study “Evaluation of the feasibility of a facility-based Test and Treat program among men who have sex with men and transgender women in Thailand (Thai Facility-based MSM/TG Test and Treat Study)”. Your responses will help us understand more about people’s attitudes towards taking anti-retroviral therapy regardless of CD4 level.
- You have the right to answer or not to answer any question. Answering or not answering will not have an negative impact on you. Your answers will help us understand your situation better.
- Your answers are confidential and will only be used for this study, and will not cause personal or legal harm.
- Some questions may make you uncomfortable or embarrassed, we apologize for this.

**Please put an “X” on the box that best describes your situation or fill in the boxes**

1. Date of birth □□/□□/□□□□ (dd/mm/yyyy)
2. How do you perceive yourself (gender identity)?

□1 Heterosexual man □2 Gay man

□3 Bisexual man □4 Transgender woman

1. How do you present yourself in public?

□1 Heterosexual man □2 Gay man

□3 Bisexual man □4 Transgender woman

1. What is your current relationship status?

□1 Single or living alone

□2 Having or living with a male partner

□3 Having or living with a female partner

1. What is your religion?

□1 Buddhism

□2 Christianity

□3 Islam

□99 Other, specify ..............................................................................

1. What level of education have you completed?

□1 Below primary school

□2 Completed primary school

□3 Completed junior high school

□4 Completed high school or vocational school

□5 Associate degree (2 years)

□6 Bachelor degree (4 years)

□7 Master degree or higher

1. What is your current occupation?

□1 Unemployed

□2 Student

□3 Self-employed / business owner

□4 Employee in private sector

□5 Day labor, driver, truck-driver

□6 Soldier/ Police

□7 Farmer

□8 Governmental staff / State Enterprise staff

□9 Sex worker in an entertainment venue/restaurant/karaoke

□99 Other ................................................

1. What is your monthly income (including salary, wages, allowance)?

□1 Below 5,000 THB □2 5,000 – 10,000 THB

□3 10,001 – 30,000 THB □4 30,001 – 50,000 THB

□5 More than 50,000 THB

1. You currently live in ..................................... province, for how many years .........?
2. You were born in .......................................................... province
3. Have you tested for HIV before this study?

□1 Never (Skip to #14)

□2 Yes, how many ______ times

1. When was the last time you tested for HIV?

□1 In the past 6 months

□2 Between 6 and 12 months ago

□3 More than 12 months ago

□4 I don’t remember

1. Where did you last test for HIV?

□1 Drop in center/ mobile service □2. Public hospital/clinic

□3. Private clinic/ Private hospital □4. Research clinic/ trial

□5. I don’t remember/ I don’t know

**THEN SKIP TO # 15**

1. Please check the reasons why you have not had an HIV test before (Check all that apply)

□1 Never thought about HIV/AIDS before

□2 I don’t belong to a risk group

□3 Fear of meeting acquaintances at VCT clinic

□4 I don’t trust the confidentiality protections at the clinic

□5 Fear of having positive result / of having AIDS

□6 Fear of rejection and stigmatization from family / partners

□7 Too busy for HIV test

□8 Fear of losing job (due to HIV positive status)

□9 Not knowing where to go for VCT

□10 Fear of needles

□11 I doubt the standard of HIV testing service at clinics

□ 12 The clinic is far and inconvenient for me to travel

□ 13 The clinic looks unclean

□ 14 The clinic does not look pleasant / nice

□ 15 Too many tedious processes for HIV testing, too time-consuming

□ 16 The clinic’s office hours are not convenient to me

□ 17 Clinic staff look unfriendly

□ 18 I don’t think I have HIV-related risks

□ 19 I don’t want to pay the testing fee

□ 99 Other (identify).................................................................

1. Do you know that you can have free HIV tests 2 times a year?

□1 Yes, I do

□2 No, I don’t

1. How did you receive information about HIV testing and this project? (Check all that apply)

□1 PDI coupon

□2. Internet/website/social network

○ 2.1 Adam’s love ○2.2 Buddystation.org ○2.3 Grindr ○2.4 Jack’d

○2.5 Hornet ○ 2.6 B-Change positive living portal ○2.7 Facebook

○2.8 SMS ○2.9 LINE ○2.10 other, specify ___________

□3. Hospital / Clinic

□4. TV / Radio

□5. Newspaper / Magazine

□6. Friend / Relative

□7. Poster / Brochure/ Postcard

□8. Peer educator / Field staff

□99. Other, specify .................................................................

1. Which of the followings would motivate you to return for your next HIV testing?

□ 1. The clinic meets national quality standards for HIV testing

□ 2. The clinic is located in a convenient spot

□ 3. The clinic is clean

□ 4. The clinic looks pleasant / nice

□ 5. The clinic has a quick process for HIV testing

□ 6. The clinic’s office hours are convenient for me

□ 7. The clinic has peer educators or outreach workers to promote its services

□ 8. The clinic staff are friendly

□ 9. Online promotions of the clinics via websites/ social network

□ 10. The logo / branding of the clinic ensures me of its quality

□ 11. Free HIV testing services

□ 12. Gifts/ souvenirs provided after I used the service

□ 99. Other, specify
