# Supplementary material for: Implementation of a Test, Treat, and Prevent HIV program among men who have sex with men and transgender women in Thailand, 2015-2016
Source: PLoS One. 2018 Jul 25;13(7):e0201171. doi: 10.1371/journal.pone.0201171 (PMC6059477; doi:10.1371/journal.pone.0201171)
Supplement: S3 File — (ZIP) [file pone.0201171.s003.zip › 2014.07.17 KPIS ARTadherenceEng.docx]

ART adherence assessment questionnaire (Approximately 5 minutes to complete)

| □ Mo 6 | □ Mo 12 | □ Mo 18 |
| --- | --- | --- |

At every doctor’s visit, we will ask you these questions to assess your adherence to anti-retroviral therapy (ART), which is very important for HIV/AIDS treatment and care. Please answer each question truthfully. We will explain to you each question and how to answer it. Then you will be asked to fill in this questionnaire. This questionnaire is adapted from the National Guideline on Monitoring and Promoting ART adherence by the Bureau of AIDS, TB and STIs, Ministry of Public Health.

1. Please rate how well you adhered to ART in the past month (Please mark the number that best reflects your adherence)

| 0% | 10% | 20% | 30% | 40% | 50% | 60% | 70% | 80% | 90% | 100% |
| --- | --- | --- | --- | --- | --- | --- | --- | --- | --- | --- |
| 0 days | 3 days | 6 days | 9 days | 12days | 15days | 18days | 21days | 24days | 27days | 30days |

Not adherent at all Very adherent

(Being adherent is defined as not missing ART and taking it within 30 minutes of the appointed time – not exceeding 30 minutes before or after the appointed time)

1. In the past week (7 days), how many days did you take ART as planned? _______ days
2. Which of the following are reasons you adhere to ART (check all that apply)

| - 1. To stay healthy and live long - 2. To suppress the virus and increase immunity, in order to avoid symptoms - 3. To reduce risk in transmitting HIV to partner(s) - 4. To be able to take care of family and the loved ones - 5. I know the disadvantages of non-adherence | - 6. Easy to take, not too many pills to take - 7. Time to take pills fits my life schedule - 8. Having supportive friends/ family or good relationship with medical staff to encourage me to take pills - 9. I did not get any negative side effects from drugs - 99. Other factors |
| --- | --- |

1. Which of the following are barriers for you to adhere to ART (check all that apply)

| - 1. I am not sure how to take the pills - 2. I am not sure of the ART benefit - 3. I don’t feel well after taking the pills - 4. Too many pills to take - 5. I lost my pills - 6. I ran out of pills / didn’t get the refill yet - 7. I decided to stop taking pills - 8. My new life schedule makes it inconvenient to take ART - 9. I shared my pills with others | - 10. I don’t want to be noticed as PLHA - 11. I forgot - 12. I didn’t know that I needed to take pills regularly and continuously - 13. I believe there will be side effects from drug (e.g., liver or renal failure) - 14. Having problem swallowing - 15. I have side effect from drug - 16. I haven’t disclosed my HIV status to family/friends so I can’t take pills openly - 99. Other factors |
| --- | --- |

1. Who helped you to adhere to ART regularly (check all that apply)

□ 1 Doctor

□ 2 Nurse

□ 3 counselor

□ 4 Peer educator/ outreach workers

□ 5 Drop-in-center staff

□ 6 Family member

□ 7 Life partner/ boyfriend

□ 8 Friends

□ 99 Others, please specify _____________________________________________
