# Supplementary material for: Implementation of a Test, Treat, and Prevent HIV program among men who have sex with men and transgender women in Thailand, 2015-2016
Source: PLoS One. 2018 Jul 25;13(7):e0201171. doi: 10.1371/journal.pone.0201171 (PMC6059477; doi:10.1371/journal.pone.0201171)
Supplement: S3 File — (ZIP) [file pone.0201171.s003.zip › 5 Qes1-PrEP accep know att version 3-30 Jan 15.docx]

□เดือน 0

**แบบสอบถามประเมินความรู้ ความต้องการและทัศนคติในการใช้ยาต้านไวรัสเพื่อป้องกันก่อนการสัมผัสเชื้อเอชไอวี**

แบบสอบถามชุดนี้ ใช้เพื่อประเมินความคิดเห็นเกี่ยวกับการรับยาต้านไวรัสเพื่อป้องกันการติดเชื้อเอชไอวีของอาสาสมัครที่เข้าร่วมโครงการ “โครงการประเมินการกินยาต้านไวรัสเพื่อป้องกันก่อนการสัมผัสเชื้อเอชไอวีในกลุ่มชายที่มีเพศสัมพันธ์กับชายและสาวประเภทสองในประเทศไทย”

- ท่านมีสิทธิที่จะไม่ตอบคำถามใดก็ได้ในแบบสอบถามชุดนี้ โดยจะไม่เกิดผลเสียใดๆ ต่อตัวท่าน อย่างไรก็ตาม ข้อมูลที่ท่านตอบจะช่วยให้เราเข้าใจลักษณะทั่วไปของท่านได้ดีขึ้น
- ข้อมูลทั้งหมดจะถูกเก็บไว้เป็นความลับ และจะนำมาใช้ในงานวิจัยเท่านั้น ข้อมูลเหล่านี้จะไม่มีผลใดๆ ทั้งสิ้นต่อตัวท่านทั้งในทางส่วนตัวและทางกฎหมาย
- คำถามบางข้ออาจจะทำให้ท่านรู้สึกไม่สบายใจ หรืออึดอัดใจ ซึ่งเราต้องขออภัยไว้ล่วงหน้า และต้องขอขอบพระคุณอย่างยิ่งที่ท่านกรุณาสละเวลาตอบแบบสอบถามชุดนี้

**การรับรู้เกี่ยวกับการกินยาต้านไวรัสเพื่อการป้องกันการติดเชื้อเอชไอวีแก่ผู้ที่ยังไม่ติดเชื้อ (ยาเพร็พ, PrEP)**

1. ท่านรู้หรือไม่ว่าการกินยาต้านไวรัสทุกวันสามารถช่วยป้องกันการติดเชื้อเอชไอวีได้

□รู้ □ ไม่รู้(ข้ามไปตอบข้อ 3) □ ไม่แน่ใจ

1. หากท่านเคยรู้เกี่ยวกับการกินยาเพร็พท่านได้รับข้อมูลมาจากที่ใด? (ตอบได้มากกว่า 1ข้อ)

□ อินเตอร์เน็ต โปรดระบุชื่อเว็บไซด์ ดังด้านล่าง

○Adam’s love ○Buddystation.org ○Gay BKK

○Facebook ของคลินิก/โครงการ ○อื่นๆระบุ______________________

□โรงพยาบาล/คลินิก □โทรทัศน์/วิทยุ

□หนังสือพิมพ์/นิตยสาร □เพื่อน/ญาติ

□อื่นๆ ระบุ.................................................................

1. หากมียาต้านไวรัสเพื่อการป้องกันการติดเชื้อเอชไอวีแก่ผู้ที่ยังไม่ติดเชื้อ(ยาเพร็พ)ท่านคิดว่าท่านจะกินไหม

□กินแน่นอน □อาจจะกิน

□อาจจะไม่กิน □ไม่กินแน่นอน □ไม่แน่ใจ

**ความรู้เกี่ยวกับPrEP**

| **กรุณาอ่านข้อความด้านล่าง และกากบาทคำตอบลงในช่อง “ใช่” หรือ “ไม่ใช่”** | **(1)**  **ใช่** | **(2)**  **ไม่ใช่** |
| --- | --- | --- |
| 1. เป้าหมายของเพร็พ คือเพื่อป้องกันการติดเชื้อเอชไอวี ในผู้ที่ยังไม่มีเชื้อเอชไอวี |  |  |
| 1. ยาที่ใช้ในเพร็พเป็นยาต้านไวรัสที่ใช้รักษาผู้มีเชื้อเอชไอวีด้วย |  |  |
| 1. เมื่อกินยาเพร็พเพื่อป้องกันแล้ว ไม่จำเป็นต้องใช้ถุงยางอนามัยอีก |  |  |
| 1. ยาเพร็พจะมีผลในการป้องกันการติดเชื้อเอชไอวีได้ดี หากกินอย่างสม่ำเสมอ |  |  |
| 1. ก่อนรับยาเพร็พท่านจำเป็นต้องตรวจเอชไอวี และมีผลตรวจเป็นลบ |  |  |
| 1. หากเริ่มกินยาเพร็พท่านไม่จำเป็นต้องมาตรวจเอชไอวีอีกต่อไป |  |  |
| 1. ในช่วงเดือนแรกของการกินยาเพร็พท่านอาจมีอาการข้างเคียงบ้างเล็กน้อย เช่นปวดหัว ปวดท้อง อาเจียน เบื่ออาหาร แต่ส่วนใหญ่อาการเหล่านี้จะหายไปเองหลังจากผ่านเดือนแรก |  |  |
| 1. ในระหว่างที่รับยาเพร็พหากมีอาการที่สงสัยว่าอาจเกิดขึ้นจากการเพิ่งได้รับเชื้อเอชไอวี เช่นเป็นไข้เจ็บคอ ปวดหัว มีผื่น ต่อมน้ำเหลืองบวม ท่านต้องรีบกลับมาตรวจเอชไอวี |  |  |
| 1. หากจะรับยาเพร็พเพื่อป้องกันการติดเชื้อ ท่านสามารถรับยาได้เลย โดยไม่ต้องมีการตรวจทางห้องปฏิบัติการใดๆอีก ยกเว้นการตรวจเอชไอวี |  |  |
| 1. ถ้าท่านลืมกินยาไปแม้เพียงหนึ่งเม็ด ท่านต้องหยุดการกินยาเพร็พไปเลย เพราะสูญเสียประสิทธิภาพในการป้องกัน |  |  |

**ข้อมูลเกี่ยวกับเพร็พ**

ปัจจุบันมีการศึกษาที่แสดงผลว่าการให้ยาต้านไวรัสในกลุ่มชายที่มีเพศสัมพันธ์กับชาย สามารถลดความเสี่ยงจากการติดเชื้อเอชไอวีในผู้ที่ยังไม่ติดเชื้อที่กินยาสม่ำเสมอควบคู่กับใช้วิธีการป้องกันการติดเชื้ออื่นๆ เช่นการใช้ถุงยางอนามัย การกินยาต้านไวรัสนี้อาจจะมีผลข้างเคียงอยู่บ้าง โดยผลข้างเคียงส่วนใหญ่ที่พบเช่นคลื่นไส้ ปวดหัว อาเจียน น้ำหนักลด เนื่องจากการกินยาต้านไวรัสเพื่อป้องกัน อาจยังไม่สามารถป้องกันการติดเชื้อเอชไอวีได้ 100% และยังไม่สามารถป้องกันการติดเชื้อโรคติดต่อทางเพศสัมพันธ์อื่นๆ ได้ผู้ที่กินยาจึงยังต้องใช้ถุงยางอนามัยควบคู่ไปด้วย และต้องมาตรวจเอชไอวีสม่ำเสมอทุก 3 เดือน

**ปัจจัยที่เกี่ยวข้องกับการตัดสินใจกินยาเพร็พและการกินยาเพร็พอย่างสม่ำเสมอ**

1. ถ้ามียาเพร็พให้กินทันที ท่านจะกินหรือไม่

□กินแน่นอน □อาจจะกิน

□อาจจะไม่กิน □ไม่กินแน่นอน

1. ถ้าต้องกินยาเพร็พทุกวัน ท่านจะกินหรือไม่

□กินแน่นอน □อาจจะกิน

□อาจจะไม่กิน □ไม่กินแน่นอน

3. ท่านจะกินยาเพร็พทุกวันหรือไม่ หากยาไม่สามารถป้องกันการติดเชื้อเอชไอวีได้ 100%

□กินแน่นอน □อาจจะกิน

□อาจจะไม่กิน □ไม่กินแน่นอน

4. ท่านจะกินยาเพร็พหรือไม่ ถ้ากินแล้วมีอาการข้างเคียงเล็กน้อย

□กินแน่นอน □อาจจะกิน

□อาจจะไม่กิน □ไม่กินแน่นอน

5. ท่านจะกินยาเพร็พหรือไม่ ถ้าท่านต้องซื้อยากินเอง

□กินแน่นอน □อาจจะกิน

□อาจจะไม่กิน □ไม่กินแน่นอน

6. หากต้องซื้อยาเพร็พมากินเอง ท่านคิดว่าราคาเท่าไรที่ท่านสามารถจ่ายได้ต่อเดือน

□ต่ำกว่า 500บาท/เดือน □500-1,000บาท/เดือน

□1,001-2,000 บาท /เดือน □ 2,001-3,000 บาท /เดือน

□ ไม่ต้องการจ่ายเงินซื้อเอง

7. ท่านจะกินยาเพร็พอยู่หรือไม่ หากยังต้องใช้ถุงยางอนามัยร่วมด้วย

□กินแน่นอน □อาจจะกิน

□อาจจะไม่กิน □ไม่กินแน่นอน

8. ท่านจะกินยาเพร็พหรือไม่ หากต้องมาตรวจเอชไอวีทุก 3 เดือนระหว่างที่กินยา

□กินแน่นอน □อาจจะกิน

□อาจจะไม่กิน □ไม่กินแน่นอน

9. หากจะกินยาเพร็พท่านอยากมารับยาที่ไหน ไม่ว่าจะรับยาฟรีหรือซื้อเองก็ตาม

□ศูนย์บริการสุขภาพในชุมชน เช่น ศูนย์ที่ท่านมาเข้าร่วมโครงการนี้

□ โรงพยาบาลที่ท่านใช้สิทธิการรักษาพยาบาลอยู่ เช่น สิทธิบัตรทอง สิทธิประกันสังคม

□ คลินิกหรือโรงพยาบาลเอกชน

□ ร้านขายยา

□อื่นๆ ระบุ _____________________________________________

10. สาเหตุที่ท่านอยากไปรับบริการยาเพร็พในสถานที่ในข้อที่ผ่านมาคือ **(ตอบได้มากกว่า 1 ข้อ)**

□**สถานที่ให้บริการได้รับการรับรองมาตรฐานในการให้บริการ**

□ **สามารถเดินทางไปยังสถานที่ให้บริการได้สะดวก**

□ **สถานที่ให้บริการมีความสะอาด**

□ **สถานที่ให้บริการมีความสวยงาม**

□**ขั้นตอนในการให้บริการมีความรวดเร็ว**

□**มีเแกนนำ/เจ้าหน้าที่ภาคสนามที่คอยช่วยเหลือ**

□ **เจ้าหน้าที่มีความเป็นมิตร เป็นกันเอง**

□ **เวลาเปิดทำการสะดวก สามารถมาตรวจได้หลังเลิกงาน/เลิกเรียน หรือในวันหยุด**

□**มีเครื่องหมายเครือข่ายสถานบริการคุณภาพ**

□ **อื่นๆ ระบุ_______________________________________________**

11.ท่านเคยมีประสบการณ์ต้องกินยาใดๆก็ตามทุกวันมาก่อนหรือไม่

□ เคย □ ไม่เคย

12.หากเคย ท่านเคยกินยาทุกวันต่อเนื่องกันเป็นเวลานานที่สุดกี่วัน _________ วัน

13.ท่านกินยาได้ครบตามกำหนดหรือไม่

□ ครบ □ ไม่ครบ

**ทัศนคติต่อการกินยาเพร็พ**

1. ท่านอายไหมที่จะกินยาเพร็พเพื่อป้องกันการติดเชื้อเอชไอวี

□อายมาก

□อาย

□ค่อนข้างอาย

□ไม่อายเลย

1. ท่านรู้สึกกังวลใจไหมถ้าจะต้องกินยาเพร็พ

□กังวลมาก

□กังวลบ้าง

□ไม่ค่อยกังวล

□ไม่กังวลเลย

1. อะไรที่ทำให้ท่านคิดว่าเป็นอุปสรรคเกี่ยวกับการกินยาเพร็พ (ตอบได้มากกว่าหนึ่งข้อ)

□ ราคา

□ผลข้างเคียงจากการใช้ยา

□ ไม่ชอบกินยา

□กลัวลืมกินยา

□กลัวครอบครัวรู้

□กลัวคู่นอนรู้

□กลัวคนรู้ว่าเป็นเกย์/สาวประเภทสอง

□กลัวคนเข้าใจผิดว่ามีเชื้อเอชไอวี

□กลัวยาไม่มีประสิทธิภาพ

□ป้องกันการติดเชื้อเอชไอวีด้วยวิธีอื่นอยู่แล้ว

□อื่นๆ ระบุ _____________________

1. ท่านรู้สึกว่าการกินยาเพร็พช่วยทำให้ท่านมีความหวังในการป้องกันเอชไอวี

□มีความหวังมาก □มีความหวังบ้าง

□ไม่ค่อยมีความหวัง □ไม่หวังเลย
