# Supplementary material for: Implementation of a Test, Treat, and Prevent HIV program among men who have sex with men and transgender women in Thailand, 2015-2016
Source: PLoS One. 2018 Jul 25;13(7):e0201171. doi: 10.1371/journal.pone.0201171 (PMC6059477; doi:10.1371/journal.pone.0201171)
Supplement: S3 File — (ZIP) [file pone.0201171.s003.zip › 6 Qes2-PrEP adherence-30 Jan 15 for EC DDC.docx]

**แบบสอบถามประเมินความต่อเนื่องในการกินยาต้านไวรัสเพื่อป้องกันการติดเชื้อเอชไอวีในกลุ่มชายมีเพศสัมพันธ์กับชายและสาวประเภทสอง**

□เดือน 1 □เดือน3 □เดือน 6 □เดือน 9 □เดือน 12

- แบบสอบถามชุดนี้ ใช้เพื่อประเมินความต่อเนื่องในการกินยาต้านไวรัสเพื่อป้องกันการติดเชื้อเอชไอวีของอาสาสมัครที่เข้าร่วม“โครงการประเมินการกินยาต้านไวรัสเพื่อป้องกันก่อนการสัมผัสเชื้อเอชไอวีในกลุ่มชายที่มีเพศสัมพันธ์กับชายและสาวประเภทสองในประเทศไทย”
- ท่านมีสิทธิที่จะไม่ตอบคำถามใดก็ได้ในแบบสอบถามชุดนี้ โดยจะไม่เกิดผลเสียใดๆ ต่อตัวท่าน อย่างไรก็ตาม ข้อมูลที่ท่านตอบจะช่วยให้เราเข้าใจลักษณะทั่วไปของท่านได้ดีขึ้น
- ข้อมูลทั้งหมดจะถูกเก็บไว้เป็นความลับ และจะนำมาใช้ในงานวิจัยเท่านั้น ข้อมูลเหล่านี้จะไม่มีผลใดๆ ทั้งสิ้นต่อตัวท่านทั้งในทางส่วนตัวและทางกฎหมาย
- คำถามบางข้ออาจจะทำให้ท่านรู้สึกไม่สบายใจ หรืออึดอัดใจ ซึ่งเราต้องขออภัยไว้ล่วงหน้า และต้องขอขอบพระคุณอย่างยิ่งที่ท่านกรุณาสละเวลาตอบแบบสอบถามชุดนี้

1. ในช่วงหนึ่งอาทิตย์ที่ผ่านมา ท่านกินยาตามที่ตั้งใจไว้กี่วัน _____ วัน
2. ท่านคิดว่าใครมีส่วนช่วยสนับสนุนให้ท่านกินยาได้อย่างสม่ำเสมอ(ตอบได้มากกว่า 1 ข้อ)

□แพทย์ □ พยาบาล

□ ผู้ให้คำปรึกษา □ แกนนำ/เจ้าหน้าที่ภาคสนาม

□ เจ้าหน้าที่ในศูนย์ □ บุคคลในครอบครัว

□ แฟน/คู่รัก □ เพื่อน

□ อื่นๆ ระบุ _____________________________________________

1. เหตุผลใดในข้อต่อไปนี้ที่ทำให้ท่านสามารถกินยาได้ต่อเนื่อง สม่ำเสมอ(ตอบได้มากกว่าหนึ่งข้อ)

□คิดว่าตนเองมีพฤติกรรมเสี่ยงต่อการรับเชื้อเอชไอวี

□เชื่อว่ายาช่วยป้องกันการติดเชื้อเอชไอวีได้

□ตารางการกินยาไม่ขัดแย้งกับตารางการใช้ชีวิตประจำวัน

□ ยากินง่าย

□ มีเพื่อน แฟน หรือครอบครัว คอยช่วยเตือนให้กินยา

□ เจ้าหน้าที่ รพ. หรือ ศูนย์เพื่อน ช่วยเตือน

□ มีอุปกรณ์ช่วยเตือนให้กินยาโปรดระบุ ______________

□เหตุผลอื่นๆ โปรดระบุ____________________________________________

1. **ในกรณีที่ท่านลืมกินยาหนึ่งวัน หรือมากกว่านั้น เหตุผลในข้อใดต่อไปนี้ที่ทำให้ท่านลืมกินยา** (ตอบได้มากกว่าหนึ่งข้อ)

□**ไม่แน่ใจว่าต้องกินอย่างไร**

□ไม่รู้ว่ายาต้องกินทุกวัน และต้องกินต่อเนื่อง

□**กินยาแล้วรู้สึกไม่ค่อยสบาย**

□**ยาหาย**

□**ยาหมด และไม่ได้มารับยาเพิ่มตามกำหนด**

□**ไม่มีพฤติกรรมเสี่ยงในช่วงที่หยุดยา**

□**ตารางชีวิตประจำวันมีการปรับเปลี่ยนทำให้ไม่สะดวกในการกินยา**

□**เอายาไปแบ่งให้คนอื่น**

□กลัวคนเห็นแล้วคิดว่ามีเชื้อเอชไอวี

□ ไม่มีเหตุผลพิเศษ แค่ลืม

□**เหตุผลอื่น นอกเหนือจากนี้ โปรดระบุ** ___________________
