# Supplementary material for: Implementation of a Test, Treat, and Prevent HIV program among men who have sex with men and transgender women in Thailand, 2015-2016
Source: PLoS One. 2018 Jul 25;13(7):e0201171. doi: 10.1371/journal.pone.0201171 (PMC6059477; doi:10.1371/journal.pone.0201171)
Supplement: S3 File — (ZIP) [file pone.0201171.s003.zip › 7 Qes3 know att-month 18-version 3-30 Jan 15.docx]

□เดือน 12

□เดือน 18

□เดือน......

**แบบสอบถามประเมินทัศนคติและความรู้หลังการใช้ยาต้านไวรัสเพื่อป้องกันก่อนการสัมผัสเชื้อเอชไอวี**

แบบสอบถามชุดนี้ ใช้เพื่อประเมินความคิดเห็นเกี่ยวกับการรับยาต้านไวรัสเพื่อป้องกันการติดเชื้อเอชไอวีของอาสาสมัครที่เข้าร่วมโครงการ “การประเมินการกินยาต้านไวรัสเพื่อป้องกันก่อนการสัมผัสเชื้อเอชไอวีในกลุ่มชายที่มีเพศสัมพันธ์กับชายและสาวประเภทสองในประเทศไทย

- ท่านมีสิทธิที่จะไม่ตอบคำถามใดก็ได้ในแบบสอบถามชุดนี้ โดยจะไม่เกิดผลเสียใดๆ ต่อตัวท่าน อย่างไรก็ตาม ข้อมูลที่ท่านตอบจะช่วยให้เราเข้าใจลักษณะทั่วไปของท่านได้ดีขึ้น
- ข้อมูลทั้งหมดจะถูกเก็บไว้เป็นความลับ และจะนำมาใช้ในงานวิจัยเท่านั้น ข้อมูลเหล่านี้จะไม่มีผลใดๆ ทั้งสิ้นต่อตัวท่านทั้งในทางส่วนตัวและทางกฎหมาย
- คำถามบางข้ออาจจะทำให้ท่านรู้สึกไม่สบายใจ หรืออึดอัดใจ ซึ่งเราต้องขออภัยไว้ล่วงหน้า และต้องขอขอบพระคุณอย่างยิ่งที่ท่านกรุณาสละเวลาตอบแบบสอบถามชุดนี้

**ความรู้เกี่ยวกับการกินยาต้านไวรัสเพื่อการป้องกันการติดเชื้อเอชไอวีแก่ผู้ที่ยังไม่ติดเชื้อ (ยาเพร็พ, PrEP)**

| **กรุณาอ่านข้อความด้านล่าง และกากบาทคำตอบลงในช่อง “ใช่” หรือ “ไม่ใช่”** | **(1)**  **ใช่** | **(2)**  **ไม่ใช่** |
| --- | --- | --- |
| 1. เป้าหมายของเพร็พ คือเพื่อป้องกันการติดเชื้อเอชไอวี ในผู้ที่ยังไม่มีเชื้อเอชไอวี |  |  |
| 1. ยาที่ใช้ในเพร็พเป็นยาต้านไวรัสที่ใช้รักษาผู้มีเชื้อเอชไอวีด้วย |  |  |
| 1. เมื่อกินยาเพร็พเพื่อป้องกันแล้ว ไม่จำเป็นต้องใช้ถุงยางอนามัยอีก |  |  |
| 1. ยาเพร็พจะมีผลในการป้องกันการติดเชื้อเอชไอวีได้ดี หากกินอย่างสม่ำเสมอ |  |  |
| 1. ก่อนรับยาเพร็พท่านจำเป็นต้องตรวจเอชไอวี และมีผลตรวจเป็นลบ |  |  |
| 1. หากเริ่มกินยาเพร็พ ท่านไม่จำเป็นต้องมาตรวจเอชไอวีอีกต่อไป |  |  |
| 1. ในช่วงเดือนแรกของการกินยาเพร็พ ท่านอาจมีอาการข้างเคียงบ้างเล็กน้อย เช่นปวดหัว ปวดท้อง อาเจียน เบื่ออาหาร แต่ส่วนใหญ่อาการเหล่านี้จะหายไปเองหลังจากผ่านเดือนแรก |  |  |
| 1. ในระหว่างที่รับยาเพร็พหากมีอาการที่สงสัยว่าอาจเกิดขึ้นจากการเพิ่งได้รับเชื้อเอชไอวี เช่นเป็นไข้เจ็บคอ ปวดหัว มีผื่น ต่อมน้ำเหลืองบวม ท่านต้องรีบกลับมาตรวจเอชไอวี |  |  |
| 1. หากจะรับยาเพร็พเพื่อป้องกันการติดเชื้อ ท่านสามารถรับยาได้เลย โดยไม่ต้องมีการตรวจทางห้องปฏิบัติการใดๆอีก ยกเว้นการตรวจเอชไอวี |  |  |
| 1. ถ้าท่านลืมกินยาไปแม้เพียงหนึ่งเม็ด ท่านต้องหยุดการกินยาเพร็พไปเลย เพราะสูญเสียประสิทธิภาพในการป้องกัน |  |  |

**ทัศนคติต่อการกินยาเพร็พ**

1. ท่านอายไหมที่จะกินยาเพร็พเพื่อป้องกันการติดเชื้อเอชไอวี

□อายมาก □อาย □ค่อนข้างอาย □ไม่อายเลย

1. ท่านรู้สึกกังวลใจไหมถ้าจะต้องกินยาเพร็พ

□กังวลมาก □กังวลบ้าง □ไม่ค่อยกังวล □ไม่กังวลเลย

1. อะไรที่ทำให้ท่านคิดว่าเป็นอุปสรรคเกี่ยวกับการกินยาเพร็พ (ตอบได้มากกว่าหนึ่งข้อ)

□ ราคา

□ผลข้างเคียงจากการใช้ยา

□ กลัวลืมกินยา

□กลัวครอบครัวรู้

□กลัวคู่นอนรู้

□กลัวคนรู้ว่าเป็นเกย์/สาวประเภทสอง

□กลัวคนเข้าใจผิดว่ามีเชื้อเอชไอวี

□กลัวยาไม่มีประสิทธิภาพ

□อื่นๆ ระบุ _____________________

1. ท่านรู้สึกว่าการกินเพร็พช่วยทำให้ท่านมีความหวังในการป้องกันเอชไอวี

□มีความหวังมาก □มีความหวังบ้าง □ไม่ค่อยมีความหวัง □ไม่หวังเลย

1. ท่านเคยมีประสบการณ์ต้องกินยาใดๆก็ตามทุกวันมาก่อนหรือไม่

□ เคย □ ไม่เคย

6. หากเคย ท่านเคยกินยาทุกวันต่อเนื่องกันเป็นเวลานานที่สุดกี่วัน _________ วัน

7. ท่านกินยาได้ครบตามกำหนดหรือไม่

□ ครบ □ ไม่ครบ

***คำถามต่อไปนี้ถามเฉพาะการมาตามนัดเดือนที่ 12***

1. หากต้องซื้อยาเพร็พมากินเอง ท่านคิดว่าราคาเท่าไรที่ท่านสามารถจ่ายได้ต่อเดือน

□ต่ำกว่า 500บาท/เดือน □ 500-1,000บาท/เดือน

□1,001-2,000 บาท /เดือน □ 2,001-3,000 บาท /เดือน

□ ไม่ต้องการจ่ายเงินซื้อเอง

***คำถามต่อไปนี้ถามเฉพาะการมาตามนัดเดือนที่ 18***

1. หากต้องซื้อยาเพร็พมากินเอง ท่านคิดว่าราคาเท่าไรที่ท่านสามารถจ่ายได้ต่อเดือน

□ต่ำกว่า 500บาท/เดือน □500-1,000บาท/เดือน

□1,001-2,000 บาท /เดือน □2,001-3,000 บาท /เดือน

□ไม่ต้องการจ่ายเงินซื้อเอง

1. ท่านได้ซื้อยาเพร็พกินเอง หลังจากหมดกำหนดรับยาฟรีจากโครงการหรือไม่

□ซื้อ โปรดระบุเหตุผล (ตอบได้มากกว่าหนึ่งข้อ)

○ยังมีความเสี่ยงอยู่

○ราคาไม่แพง

○หาซื้อง่าย

○มีประโยชน์

○ไม่มีอาการข้างเคียง

○อื่นๆ โปรดระบุ _____________________

□ไม่ได้ซื้อ โปรดระบุเหตุผล (ตอบได้มากกว่าหนึ่งข้อ)

○ไม่มีความเสี่ยงแล้ว

○ราคาแพง

○หาซื้อยาก

○ไม่เห็นประโยชน์

○มีอาการข้างเคียง

○อื่นๆ โปรดระบุ _____________________
