# Supplementary material for: Implementation of a Test, Treat, and Prevent HIV program among men who have sex with men and transgender women in Thailand, 2015-2016
Source: PLoS One. 2018 Jul 25;13(7):e0201171. doi: 10.1371/journal.pone.0201171 (PMC6059477; doi:10.1371/journal.pone.0201171)
Supplement: S3 File — (ZIP) [file pone.0201171.s003.zip › KPIS ART knowledge attitudes.pdf]

Participant ID: ☐☐☐☐
Date : ☐☐/☐☐/☐☐☐☐

Questionnaire: HIV knowledge and attitudes towards ART use regardless of CD4 count (approximately 5 minutes)

VISIT: ☐ **Entry visit** (before HIV test)
☐**Wk.0** (after HIV test, counseling for ART)
☐ **Mo. 6** (if negative or not receiving ART)
☐ **Mo.12** (if negative or not receiving ART)
☐ **Mo.18** (if negative or not receiving ART)

- This questionnaire aims to collect information from participants in the study "Evaluation of the feasibility of a facility-based Test and Treat program among men who have sex with men and transgender women in Thailand (Thai Facility-based MSM/TG Test and Treat Study)". Your responses will help us understand more about people's attitudes towards taking anti-retroviral therapy regardless of CD4 level.
- You have the right to answer or not to answer any question. Answering or not answering will not have an negative impact on you. Your answers will help us understand your situation better.
- Your answers are confidential and will only be used for this study, and will not cause personal or legal harm.
- Some questions may make you uncomfortable or embarrassed, we apologize for this.

Please put an "X" on the box that best describes your understanding/ current situation

1. HIV can be transmitted by which of the following routes? (check all that apply)
   ☐1 Vaginal or anal intercourse
   ☐2 Sharing meals with people who have HIV
   ☐3 Receiving blood / or blood-related transfusion
   ☐4 Sharing injecting equipment with people who have HIV
   ☐5 Kissing
   ☐6 Using the same bathroom as people who have HIV
   ☐7 Taking care of people who have HIV
   ☐8 Mosquito bites
   ☐9 From a mother who has HIV to her child during pregnancy
   ☐99 Other (specify)..

2. Which of the following can prevent or reduce your risks of getting HIV? (check all that apply)
   ☐1 Sexual abstinence
   ☐2 Withdrawal before ejaculation
   ☐3 Correct use of condoms at every sexual intercourse
   ☐4 Choosing a sex partner that looks healthy
   ☐5 Always cleaning your anus and genital area after sexual intercourse
   ☐6 Being circumcised or only having intercourse with circumcised men
   ☐99 Other (specify)..

3. Do you know anyone (friends/ relatives/ partners/ colleagues, etc.) who has HIV?
   ☐1 Yes, I do
   ☐2 No, I don't
   ☐3 I'm not sure

☐4 I don't want to answer

4. How do you feel about people who have HIV? (check all that apply)
   ☐1 I don't want to be around them because of the risk of HIV transmission
   ☐2 I am disgusted by their activities and probably lead to HIV infection
   ☐3 I don't feel one way or the other. We can live together.
   ☐4 I sympathize with them
   ☐5 I want to help them
   ☐99 Other, please specify ....

5. Have you received information about anti-retroviral drugs before?
   ☐1 Yes, I have
   ☐2 No, I have not (skip to #7)
   ☐3 I'm not sure (skip to #7)

6. From what source did you receive the information? (check all that apply)
   ☐1 Internet
   ☐2 Hospital / Clinic
   ☐3 TV / Radio
   ☐4 Newspaper / Magazine / Books
   ☐5 Peer educators
   ☐6 Friends / Relatives
   ☐7 Other, please specify ....

7. Who do you think should start ART?
   ☐1 All people who have HIV/AIDS
   ☐2 Only HIV positive persons who have low immunity or HIV related infections
   ☐3 Only HIV positive persons who have HIV-uninfected sexual partners, or partners who do not know their HIV status
   ☐4 I'm not sure
   ☐5 Other, specify____

8. According to you, what is/are you most concerned about if you take ART? (check all that apply)
   ☐1 I'm not concerned about any side effects
   ☐2 Lipodystrophy (sunken cheeks, thin arms/legs and potbelly)
   ☐3 Anemia
   ☐4 Kidney problems
   ☐5 Liver problems
   ☐6 Rash (medicine allergies)
   ☐7 Having to take the medicine at the same time, every day, for life
   ☐8 Cost of medicine and treatments
   ☐9 My health plan (if it covers everything, under what conditions)

□10 What my family will think if they find out I am taking ART
□11 What my friends will think if they find out I am taking ART
□12 Other (specify)..

9. Are the following statements true or false? Please select true or false.

	True	False
9.1 ART, in addition to keeping us healthy and alive, can keep us free from opportunistic infections, coronary heart disease, liver disease, and kidney disease?		
9.2 ART will also lower the risk that someone who is infected with HIV will transmit the virus to the person you have sex with, who may be your partner.		

10. If you received an HIV positive result today, or if you already knew you were HIV infected, would you start antiretroviral medicine? Given that the doctor/nurse agreed that you start the medicine and would provide routine follow-up free of charge.
□1 Yes, I would
□2 No, I would not
□3 I'm not sure
